# Positive Regadenoson Stress Test in a Patient on Phentermine With Normal Coronaries

**DOI:** 10.7759/cureus.32138

**Published:** 2022-12-02

**Authors:** Selin Sendil, Medeona Gjergjindreaj, Matthew Lozier, Rafle Fernandez

**Affiliations:** 1 Cardiology, Mount Sinai Medical Center, Miami, USA

**Keywords:** coronary artery vasospasm, raynaud’s phenomenon, regadenoson, nstemi, non-st elevation myocardial infarction, phentermine

## Abstract

Obesity is a well-established cardiovascular (CV) risk factor with greater mortality and morbidity rates than the general population. Phentermine is a weight loss medication that is approved for short-term obesity treatment in conjunction with lifestyle modifications to decrease CV risk.

A 51-year-old female with Raynaud’s phenomenon who was started on phentermine one week prior presented with a one-day history of palpitations. Subsequent workup revealed non-ST elevation myocardial infarction (NSTEMI) on presentation and worsening ST segment depressions following regadenoson injection during pharmacological stress testing.

Although current evidence suggests that the use of phentermine is safe and may even reduce the risk of CV disease in obese patients, it still may pose adverse CV effects. A detailed medical history, including medications used and predisposing conditions, is crucial to help identify and possibly prevent exacerbation of such CV side effects.

## Introduction

Obesity is a known risk factor for cardiovascular (CV) morbidity and mortality. Phentermine is an amphetamine-related weight loss medication approved by the Food and Drug Administration (FDA) for the treatment of obesity in conjunction with diet and exercise. It is generally indicated for patients with body mass index (BMI) ≥ 30 kg/m² or ≥27 kg/m² in the presence of other traditional CV risk factors such as hypertension, diabetes, and hyperlipidemia. Potential CV side effects of this medication have been reported in the literature [1]. We present a case of a patient recently started on phentermine who presented with non-ST elevation myocardial infarction (NSTEMI) and developed worsening ST segment depressions following regadenoson injection.

## Case presentation

**Figure 1:**
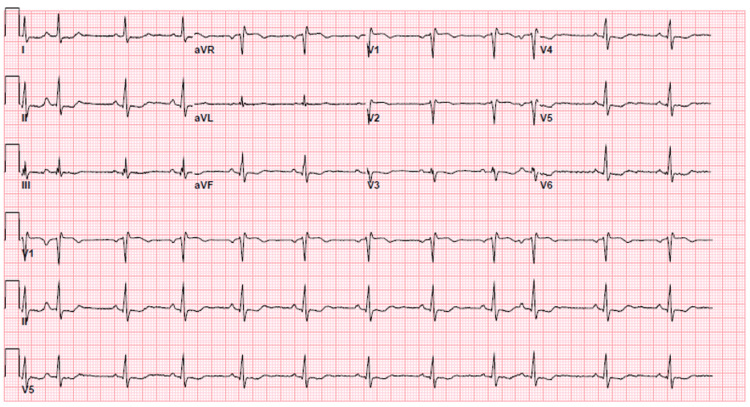
ECG on arrival to the emergency room showing sinus arrhythmia, incomplete RBBB, and TWI in anterolateral and inferior leads. ECG: electrocardiogram; RBBB: right bundle branch block; TWI: T-wave inversion

A 51-year-old female with a past medical history of hyperlipidemia, Raynaud’s phenomenon, overweight with a BMI of 27 kg/m², adrenal adenoma, hepatic angioma, psoriasis, and migraines presented to the emergency department (ED) with persistent palpitations starting one day prior to arrival. The week prior to her presentation, she had been started on phentermine for weight loss. The last dose of the medication was six hours prior to presentation. In the ED, her temperature was 36.7°C, blood pressure was 150/104 mmHg, heart rate was 64 beats/minute, respiratory rate was 18 breaths/minute, and 98% oxygen saturation on room air. Her physical examination was unrevealing. Her initial laboratory workup was unremarkable, except for mildly elevated high-sensitivity troponin of 100 pg/mL (reference range: 4-54 pg/mL). Her 12-lead electrocardiogram (ECG) showed new T-wave inversions (TWI) in anterolateral and inferior leads (Figure 1), which were not present on a prior ECG (not shown).

**Figure 2:**
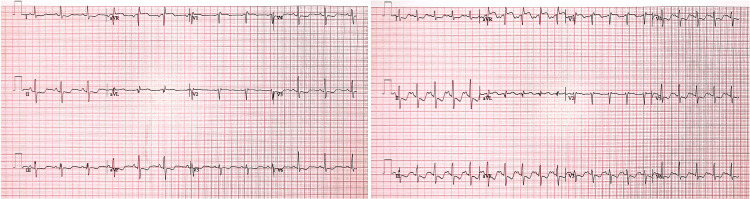
ECG recordings of the patient were shown at baseline (on the left) and after the regadenoson injection (on the right) during radionuclide MPI. New ST segment depressions following regadenoson injection can be seen in the ECG tracing on the right. ECG: electrocardiogram; MPI: myocardial perfusion imaging

Continuous telemetry monitoring and repeat ECG revealed no significant arrhythmias or tachycardia despite ongoing symptoms of palpitations. Transthoracic echocardiogram (TTE) revealed an ejection fraction (EF) of 55% without any regional wall motion abnormalities, preserved right ventricular function, and no evidence of valvular pathology. A decision was made to proceed with radionuclide myocardial perfusion imaging (MPI) with regadenoson stress testing. Upon intravenous injection of regadenoson (0.4 mg over 10 seconds), the patient complained of hand cramping. The ECG portion of the test showed worsening ST segment depressions in inferior and anterolateral leads, suggestive of ischemia (Figure 2). EF was reported as 69% in MPI.

**Figure 3:**
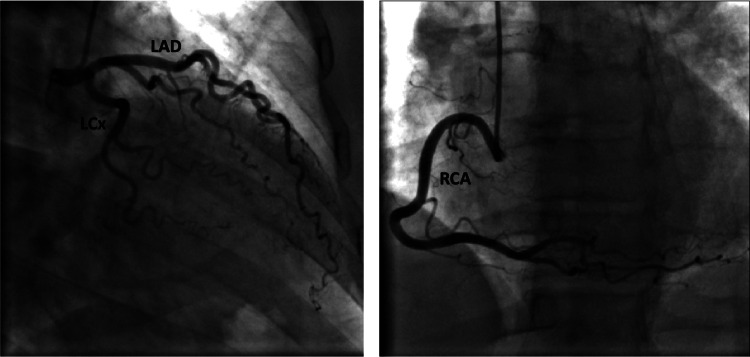
Cardiac catheterization showing angiographically normal major epicardial coronary arteries (LAD, LCx, and RCA). LAD, left anterior descending artery; LCx; left circumflex artery; RCA, right coronary artery

Nuclear stress images showed no scintigraphic evidence of myocardial ischemia; however, due to significantly positive ECG findings concerning for ischemia during the stress test, a decision was made to proceed with coronary angiography, which subsequently revealed angiographically normal epicardial coronaries (Figure 3).

**Figure 4:**
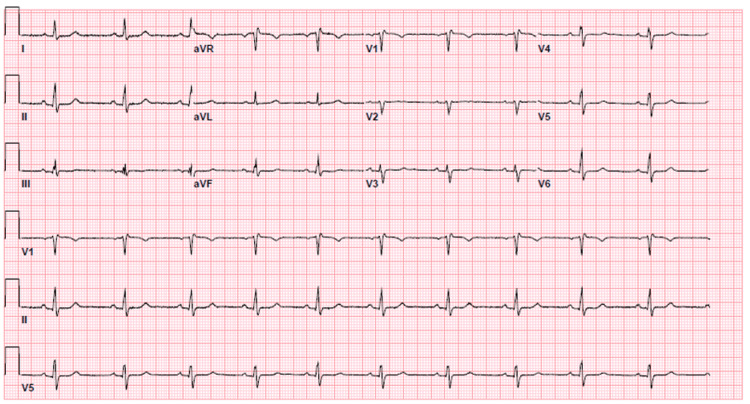
ECG during follow-up in the office visit 18 days after hospitalization. ECG: electrocardiogram

The patient was seen in the office in a few weeks following discharge from the hospital. Her ECG at the time was back to her baseline (Figure 4), and she did not have any recurrence of symptoms off phentermine in the interim.

## Discussion

Phentermine is a sympathomimetic stimulant that is chemically similar to amphetamine and is believed to increase heart rate and blood pressure [1]. The side effects of phentermine include tachycardia, hypertension, dry mouth, anxiety, insomnia, palpitations, and constipation [1]. Coronary vasospasm and subsequent myocardial infarction (MI) in patients on phentermine have been reported, as well as infrequent reports of ischemic stroke, myocardial infarction, ventricular arrhythmia, and cardiac arrest [2-5]. Our patient’s presenting symptom to the ED was palpitations without evidence of significant arrhythmia. Her palpitations may perhaps represent symptoms of coronary vasospasm rather than arrhythmia.

Adenosine and regadenoson are widely used as pharmacological agents for MPI. Regadenoson is a newer pharmacological stress agent with a higher selectivity for the A2A adenosine receptor and fewer side effects than adenosine [6]. Although the precise mechanism is unknown, reports of coronary vasospasm and myocardial infarction in some cases with both agents have been described [7,8]. The Food Drug Administration (FDA) reviewed the FDA Adverse Event Reporting System (FAERS) database for cases of myocardial infarction (MI) and death from all causes associated with Lexiscan (regadenoson). FAERS data for Lexiscan from June 24, 2008, to April 10, 2013, contained 26 cases of MI and 29 cases of death [9]. Similarly, phentermine has also been associated with coronary vasospasm. It is hypothesized that increased coronary sensitivity and predisposition to coronary vasospasm might be responsible [5].

Our patient’s presentation with palpitations and mild troponin elevation may have been related to her recent initiation of phentermine. When she underwent MPI with regadenoson, the ECG portion of the stress test was concerning for ischemia; however, coronary angiography revealed no significant obstructive coronary artery disease, raising suspicion for possible coronary vasospasm resulting in transient regadenoson-induced electrocardiographic changes.

Although CV events with the individual use of phentermine or regadenoson have been reported, to our knowledge, interactions with the concomitant use of these two agents have not been described. We believe that our patient’s clinical course may represent a possible synergistic or potentiating effect of both medications. Our patient presented with NSTEMI and palpitations in the absence of significant tachycardia or arrhythmias. Her hand cramping and significant ST depressions following injection of regadenoson could, in part, be explained by coronary vasospasm. Her recent initiation of phentermine and known history of Raynaud’s phenomenon may have further predisposed her to such events. We believe that in our patient, both her use of phentermine and subsequent administration of regadenoson likely contributed to her clinical course.

## Conclusions

Although current evidence suggests that phentermine is safe and may even reduce the risk of CV disease in obese patients, it is not free of adverse CV effects. Given the growing prevalence of obesity and the potential use of this drug, clinicians should be aware of such adverse events. It is vital to maintain a high index of suspicion for coronary vasospasm in patients presenting with evidence of myocardial infarction without obstructive coronary artery disease or anomalies. Phentermine may have vasospastic properties, which are essential when prescribed to patients. Furthermore, one should be aware of potentiating effects of other therapies. A detailed medical history, including medications used and predisposing conditions, is crucial to help identify and possibly prevent exacerbation of such CV side effects.
